# Carbon: nitrogen stoichiometry following afforestation: a global synthesis

**DOI:** 10.1038/srep19117

**Published:** 2016-01-08

**Authors:** Xia Xu, Dejun Li, Xiaoli Cheng, Honghua Ruan, Yiqi Luo

**Affiliations:** 1 Co-Innovation Center for Sustainable Forestry in Southern China, College of Biology and the Environment, Nanjing Forestry University, Nanjing, Jiangsu Province, 210037, China; 2 Department of Microbiology and Plant Biology, University of Oklahoma, Norman, OK, 73019, USA; 3 Institute of Subtropical Agriculture, Chinese Academy of Sciences, Changsha, Hunan, 410125, China; 4 Key Laboratory of Aquatic Botany and Watershed Ecology, Wuhan Botanical Garden, Chinese Academy of Sciences, Wuhan, 430074, China

## Abstract

Though carbon (C): nitrogen (N) stoichiometry has been widely studied in terrestrial ecosystems, little is known about its variation following afforestation. By synthesizing the results of 53 studies, we examined temporal and spatial variation in C: N ratios and in N-C scaling relationships of both the organic and the mineral soil horizons. Results showed that C: N ratios remained constant in the mineral horizon but significantly decreased in the organic horizon over the age sequence following afforestation. Among different climate zones, C: N ratios of the organic and the mineral horizons increased and decreased, respectively, with increasing mean annual temperature (MAT) (decreasing latitude). Pasture exhibited higher C: N ratios than cropland in the organic horizon while C: N of the mineral horizon did not change much among different land use types. For both the organic and the mineral horizons, hardwoods exhibited lower C: N ratios than pine and softwoods. Additionally, N and C in general scaled isometrically in both the organic and the mineral horizons over the age sequence and among different climate zones, land use types, and plantation species following afforestation. Our results suggest that C and N may remain coupled following afforestation.

The elements carbon (C), nitrogen (N), and phosphorus (P) are critical to all biological processes. In 1958, Redfield observed a well-constrained C: N: P ratio of C_106_:N_16_:P_1_ in marine plankton^1^. Though the Redfield ratio was originally used as an indicator of the mass balance of C, N, and P required by marine plankton^2^,^3^, it has proven to be valuable in understanding the importance of the relative proportions between elements for biological processes and nutrient cycling in marine ecosystems^1^,^4^. The Redfield ratio has influenced our knowledge on carbon dioxide exchange between oceans and the atmosphere^5^, the extent of nutrient limitation of net primary productivity^6^,^7^, and biogeochemical cycles in marine ecosystems^8^,^9^. Moreover, a new discipline, ecological stoichiometry, has come into being in ecology, which aims to understand the constraints and consequences of the mass balance of multiple chemical elements in ecological interactions with their environments from the individual organisms to the ecosystem level^4^,^10^.

The Redfield ratio is one of the most powerful and useful principles in understanding chemical composition and biogeochemical cycles of elements in marine ecosystems^4^,^11^, by which ecologists have been inspired to search for similar patterns in terrestrial ecosystems^4^,^11^,^12^,^13^. Though the element concentrations of different ecosystem components may vary, on average, atomic C: N: P ratios for the foliage (1212: 28: 1)^12^, litter (3007: 45: 1)^12^, mineral soils (186: 13: 1)^4^, and microbes (60: 7: 1)^4^ are well-constrained globally. Also, constrained C: N ratios exist in litter, forest floor, and mineral soils during stand development in forest ecosystems^11^. Moreover, previous studies indicated that parallel interactions between organisms and their environments exist in terrestrial plant communities and forest ecosystems at a global scale^11^,^12^,^13^. For example, Reich and Oleksyn^13^ revealed that the N: P ratio of plant foliage increases from high to low latitudes with the increasing average temperature and growing season length. Additionally, N scaled isometrically with C (i.e. the slope of the relationship between log N and log C is not significantly different from 1.0) in different ecosystem components, such as mineral soils^4^, foliage^12^ and in litter, forest floor, and mineral soils during forest stand development^11^. These and many other studies have provided a key to our understanding of C: N: P stoichiometry temporally and spatially in terrestrial ecosystems. However, considerable uncertainty still surrounds the variation in C: N ratios and whether the isometric relationship exists between N and C following afforestation.

Many studies have examined the dynamics of C and N stocks following afforestation^14^,^15^,^16^,^17^, which provide an ideal opportunity and makes it possible to quantify the temporal and spatial patterns of C: N stoichiometry in terrestrial ecosystems. Afforestation, a critical element of land use change, is believed to be a cost-effective option to mitigate the build-up of atmospheric carbon dioxide and enhance biospheric C stocks^14^,^18^. Globally, the area of planted forests is as large as 139.1 million hectares in 2005^19^. Knowledge of C: N stoichiometry following afforestation could advance our understanding of the effectiveness of C sequestration via afforestation and provide support for policy-making. Currently, the effects of afforestation on soil C sequestration appear to vary greatly. Increases^16^,^20^, no changes^15^ and decreases^17^,^21^ in soil C stocks have been reported. One of the possible reasons for the inconsistency is that we have neglected the C-N interactions that are important in determining whether the C accumulation in soil is sustained over time^22^,^23^. Additionally, the temporal variability of C: N stoichiometry is much less studied compared to its spatial patterns^24^. We know little about whether the C: N ratio is constrained through time following afforestation, and whether the isometric pattern between N and C exist temporally. It has been suggested that spatial ecological patterns do not necessarily occur through time^25^,^26^.

In this study, we investigated the temporal and the spatial patterns of the C: N ratio in the organic and the mineral soil horizons over the age sequence and among climate zones following afforestation, respectively, by synthesizing data extracted from 53 peer-reviewed papers. Additionally, we compared the C: N ratio among different land use types and plantation species. We also examined the stoichiometric relationship between N and C after afforestation under the four conditions mentioned above. Specifically, we aimed to test: (1) Does the C: N ratio remain relatively constant over the age sequence and among different climate zones, land use types, and plantation species after afforestation? and (2) If the C: N ratio varies, does N scale isometrically with respect to C following afforestation?

## Materials and Methods

### Data compilation

We collected data from 53 published papers (of which 16 reported organic layer data and 41 reported mineral layer data) from the literature that present both soil C and N dynamics after afforestation, or could be calculated based on percentage contents of C and N (Table S1). The following criteria were used to select the papers: both soil C and N stocks were presented or could be calculated according to percentage contents of C and N, bulk density, and sampling depth; the experiments used pair-site, chronosequence, or retrospective design, with similar soil conditions for both afforested and prior land use sites; years since afforestation were either clearly pointed out or could be directly derived; studies reporting short-term effect of afforestation (<5 yr) were excluded; only afforestation of the first rotation was considered. Additionally, studies were rejected from the data compilation if they were subject to a lack of replications, or if the paired sites or the sites of chronosequence were confounded by different soil types. The raw data were obtained from published tables or extracted from published graphs using the software Get Data Graph Digitizer (Version 2.24, Russian Federation). Data from studies that sampled many replicate plots over a single landscape, plots with the same age, edaphic conditions, and land use type were pooled together. When more than one soil depth was sampled, C and N stocks from all the depths were summed together. For the studies using chronosequence design, each age was regarded as an independent study and the data was included in the analysis. The final dataset was separated into two subsets, the organic and the mineral horizons. Ages for the plantation after afforestation were divided into three groups (5–20, 20–50, and ≥50 years) for the organic soil horizon due to the limited number of observations, and into six groups for the mineral soil horizon (5–10, 10–20, 20–30, 30–40, 40–50, and ≥50 years). Land use types prior to afforestation were categorized into cropland, natural grassland, and pasture. Climate zones were categorized into boreal, temperate continental, temperate maritime, subtropical, and tropical based on Köppen’s classification^18^. Tree species planted were classified as pine, eucalyptus, hardwoods (excluding eucalyptus), and softwoods (excluding pine).

To increase the comparability of data derived from different studies, we converted the original soil C or N into soil C or N stocks in the top 100 cm using the depth functions developed by Jobbágy and Jackson^27^,^28^:


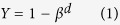



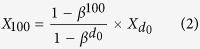


where *Y* represents the cumulative proportion of the soil C (or N) stock from the soil surface to the depth *d* (cm). *β* is the relative rate of decrease in the soil C (or N) stock with soil depth. *X*_100_ denotes the soil C (or N) stock in the upper 100 cm (g m^−2^). *d*_0_ is the original soil depth available in individual studies (cm). *X*_d0_ is the original soil C (or N) stock (g m^−2^). In this study, the global average depth distributions of C and N were applied to estimate *β* (0.9786 for C and 0.9831 for N) in Equation (Eqn. 1)^14^. The soil C (or N) stock in the upper 100 cm could then be estimated from the original soil C (or N) data using Eqn. 2. The same method (i.e. converting the original C and N stocks to the stocks in the top 100 cm using the depth functions in order to increase comparability) was used by Yang *et al.*^29^ and they concluded that depth correction did not alter the overall pattern of soil C and N stock dynamics during stand development. The analyses of our data showed that there was no significant difference between the measured and calculated values for C or N stocks^14^.

### Data analysis

We processed the data through the following two steps. First, we examined the C: N ratio to test whether it remains constant over the age sequence and to investigate the potential differences among different climate zones, land use types, and plantation species after afforestation with one-way ANOVA (due to limited and unbalanced data among groups). Relationships of C: N ratios with the age and climate zones as indicated by mean annual temperature (MAT) and latitude were analyzed with linear regression. These statistical analyses were conducted using SPSS 17.0 for windows (SPSS Inc., Chicago, IL, USA). Second, we explored the N-C relationship to test whether an isometric relationship between N and C (i.e. the slope of the relationship between log N and log C is not significantly different from 1.0) occurred through time and among different climate zones, land use types, and plantation species following afforestation. In comparison to the C: N ratio, the N-C relationship could indicate the proportional changes between N and C stock. To do this, we performed reduced major axis (RMA) regression to examine the C – N relationships. We used a log-log function^4^,^12^,^29^ to quantify the stoichiometric relationship between N and C:


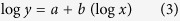


where x is C stock (g m^−2^), y is N stock (g m^−2^), a is the intercept, and b is the scaling slope. The scaling slope and *y*-intercept of the allometric function were determined using the software package “Standardized Major Axis Tests and Routines”^30^. If the slope of the N-C stoichiometric relationship was not significantly different from 1.0 (i.e. the 95% of confidence interval of the slope covered 1.0), the isometric hypothesis held true.

## Results

**Figure 1:**
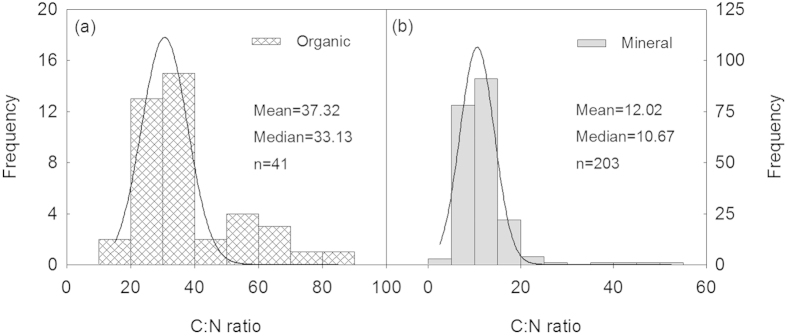
Frequency distributions of the C: N ratio in the organic (**a**) and the mineral (**b**) soil horizons. The solid curves are Gaussian distributions fitted to the frequency data.

**Figure 2:**
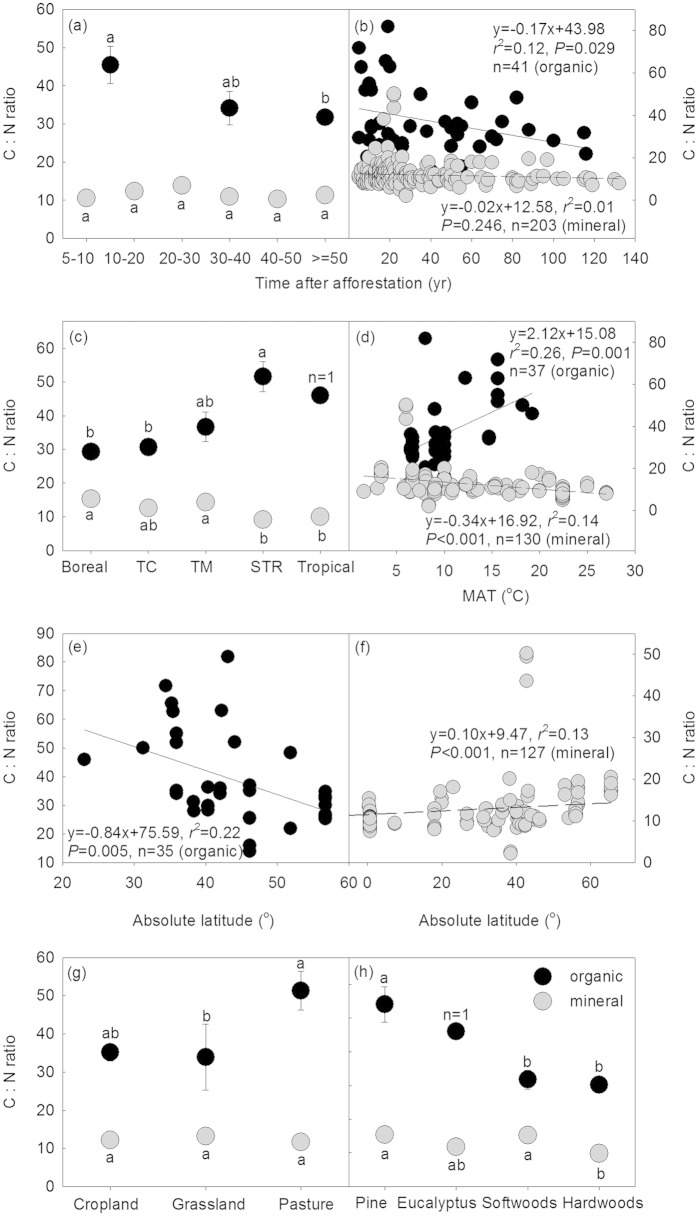
Comparison of the C: N ratio over the age sequence (a) and among different climate zones (c), land use types (g), and plantation species (h) following afforestation and relationships of C: N ratio with ages after afforestation (b), mean annual temperatures (MAT, (d), and latitudes (e,f). For the organic soil horizon in panel (**a**), the data were grouped into 5–20 yr, 20–50 yr, and ≥50 yr due to the limited number of observations. Different letters indicate statistically significant difference at *P* < 0.05 (Turkey test). Values are Mean ± SE except for the organic horizon of the tropical zone in panel (**c**) (n = 1) and the eucalyptus in panel (**f**) (n = 1). TC: temperate continental; TM: temperate maritime; STR: subtropical. The exact number of observations for each data point could be found in Fig. 3 of this study.

The C: N ratios varied greatly following afforestation (Fig. 1). The mean C: N ratio of the organic and the mineral horizons was 37.32 ± 2.37 and 12.02 ± 0.41, respectively. Generally, variation in C: N ratios of the organic and the mineral soil horizons showed remarkable differences over the age sequence and among different climate zones, land use types, and plantation species. The C: N ratio of the organic horizon significantly decreased (*P* < 0.05) while the C: N ratio of the mineral soil horizon remained constant over the age sequence following afforestation (*P* > 0.05, Fig. 2a,b). Among different climate zones, C: N ratios of the organic horizon increased and decreased, for the organic and the mineral horizons respectively, from boreal and temperate zones to tropical and subtropical zones (Fig. 2c). Further analyses showed that the C: N ratio of the organic horizon increased with increasing mean annual temperature (MAT) (*P* < 0.01, Fig. 2d) and decreased with increasing latitude (*P* < 0.01, Fig. 2e). In contrast, C: N ratios of the mineral horizon decreased with increasing MAT (*P* < 0.01, Fig. 2d) and increased with increasing latitude (*P* < 0.05, Fig. 2f). Additionally, C: N ratios of the organic and the mineral soil horizons varied among land use types before afforestation and plantation species (Fig. 2g,h). For example, pasture exhibited higher C: N ratios than cropland (Fig. 2g) while C: N of the mineral soil horizon did not experience significant changes among different land use types. For both the organic and the mineral horizons, hardwoods exhibited lower C: N ratios than pine and softwoods (Fig. 2h).

**Figure 3:**
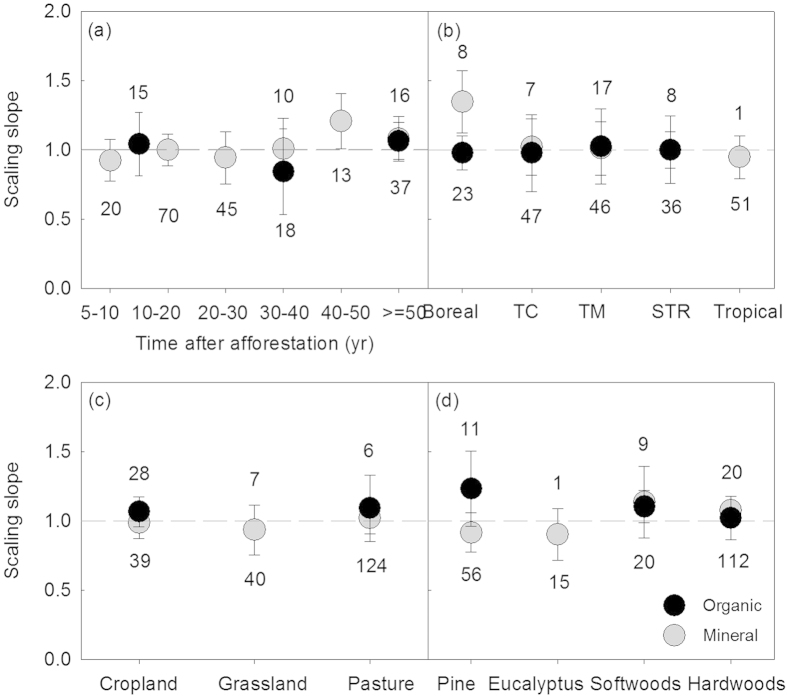
Variation in the scaling slopes of the N-C stoichiometric relationships over the age sequence (a) and among different climate zones (b), land use types (c), and plantation species (d) following afforestation. For the organic horizon in panel (**a**), the data were grouped into 5–20, 20–50, and ≥50 yr due to the limited number of observations. The scaling slope indicates the slope of the type II (i.e. reduced major axis, RMA) relationship between log-transformed N and C. The error bar shows the 95% confidence interval of the scaling slope of the relationship between log N and log C. The dash line denotes the scaling slope is equal to 1.0. The missing value in panel (**c**) for the grassland was due to *r*^2^ = 0.33 and *P* = 0.18 > 0.05 of the correlation between the log-transformed N and C but in panel (**d**) was because of the limited number of observations for the organic horizon. The values showed above and below the 1.0 line in each panel represent the number of observations for the organic and the mineral soil horizons, respectively. TC: temperate continental; TM: temperate maritime; STR: subtropical.

**Figure 4:**
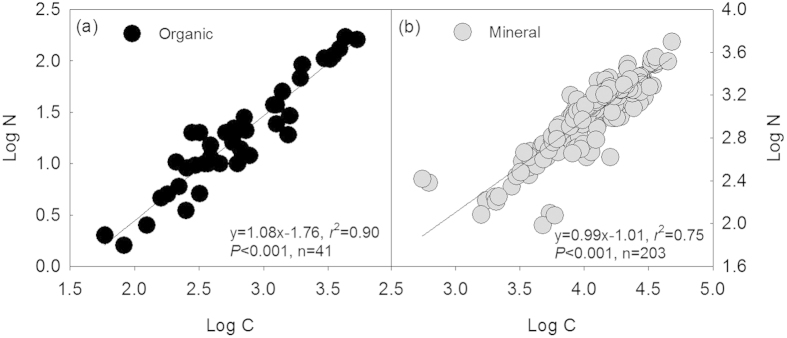
N-C stoichiometric relationships for the organic (a) and the mineral (b) soil horizons.

The scaling slopes of the organic and the mineral soil horizons did not reveal significant differences either over the age sequence, or among different climate zones, land use types, and plantation species following afforestation (Fig. 3). Generally, the allometry of N with respect to C (i.e. the slope of the N-C stoichiometric relationship between log N and log C) was predictable and constant, and not statistically different from 1.0 (*P* > 0.05), except for the boreal region where the scaling slope was significantly larger than 1.0 (Fig. 3b). The stoichiometric relationship between N and C of the organic and the mineral soil horizons was different (Fig. 4). However, N and C exhibited isometric relationships both in the organic and the mineral soil horizons following afforestation (Table 1; Fig. 4).

## Discussion

### Variation in the C: N ratio after afforestation

In line with previous studies^11^,^31^,^32^, our results showed that the C: N ratio of the mineral soil horizon in general was much smaller than that of the organic horizon (Fig. 1). In comparison to the mean C: N ratio of 37.32 ± 2.37 for the organic horizon, the mean C: N ratio of 12.02 ± 0.41 for the mineral horizon is much closer to the well-constrained global mean C: N ratio of 8.57 for microbes^4^, suggesting that organic matter in the mineral soil horizon has been assimilated more than in the organic horizon by microbes. The temporal C: N ratio dynamics of the organic horizon significantly decreased with increasing years of afforestation (Fig. 2a,b). This is probably because total N accumulation increases due to the increased biomass with increasing stand age^33^. Moreover, as the decomposition process progresses, C: N ratio always decreases because of the microbial utilization of the most easily degraded plant compounds and the gradual accumulation of the more recalcitrant materials in the organic soil horizon^34^. Previous study has revealed that most naturally occurring compounds could completely be mineralized into inorganic forms through microbial assimilation, and only approximately 7% of the detritus would eventually be stabilized into humus^35^,^36^, which explains the relatively constant C: N ratio in the mineral horizon over the age sequence after afforestation (Fig. 2a,b).

N: P ratio variation in relation to latitude or temperature globally^13^ and across Europe^37^ inspired us to examine whether any relationships exist between C: N ratios after afforestation with latitude and temperature. Our results showed that C: N ratios of the organic and the mineral soil horizons decreased and increased, respectively, with increasing latitude (decreasing temperatures, Fig. 2d–f), coincident with biogeographical gradients of climate. Climate could impact C: N ratios via biotic processes associated with plant productivity and organic matter decomposition^38^,^39^. Along with the decreasing latitude, ecosystems are gradually characterized by larger litter production and more rapid decomposition^39^,^40^, making it hard to find the organic horizon in tropical regions (n = 1 for the tropical, Fig. 2c) except in places of high elevation. Comparing with the boreal regions, fast litter input and turnover in tropical regions leads to high C: N ratio in the organic soil horizon. On the other hand, C: N ratio of the mineral horizon is low in the tropical as labile organic matter is preferentially degraded before incorporating into mineral soil. Temperature, by which plant productivity and organic decomposition are strongly impacted^41^,^42^, is projected to increase another 0.3 to 4.8 °C globally for 2081–2100 in relative to 1986–2005^43^. These patterns of C: N ratio with temperature and latitude could thus change due to complex stoichiometric responses to perturbation between C and N cycles^44^,^45^. Therefore more study of variation in soil C: N ratio following afforestation in response to climate change is warranted.

In general, previous land use types did not significantly affect C: N ratios much following afforestation (Fig. 2g). For the organic horizon, however, C: N ratio of pasture was larger than the ratios of cropland and grassland. This is attributable to the plantation species planted because aboveground biomass in cropland, grassland, and pasture is usually periodically removed by harvest, fires^46^, and grazing. According to our results, C: N ratio of pine was the highest compared to eucalyptus, softwoods, and hardwoods (Fig. 2f). Our datasets showed that pine was planted in 67% of the studies conducted in previous pasture, while the proportions for cropland and grassland were only 18% and 29%, respectively. Surprisingly, the high C: N ratio of pasture in the organic horizon did not result in a higher C: N ratio in the mineral horizon of pasture than that of cropland and grassland (Fig. 2g). This may result from the effects of pine on C: N ratio was masked by other species. Moreover, decomposition, including fragmentation, leaching of water-soluble compounds, and microbial catabolism, is an essential ecological process^47^,^48^. As decomposition progresses, the organic matter transformed into mineral soil becomes more and more similar among different land use types due to the degradation of labile organic matter and the formation of humus^34^,^49^. Additionally, pine exhibited highest C: N ratio among different species (Fig. 2h), which may be related to variations in litter quality. This is in line with previous chemical analyses, indicating that the amount of substrates with a relatively high C: N ratio, such as cell wall polysaccharide, hemicellulose, and lignin, is higher in pine than in hardwoods^34^. Interestingly, C: N ratios in the mineral horizon of softwoods was higher than that of hardwoods (*P* < 0.05), though the C: N ratio in the organic horizon of the two species did not differ from each other (*P* > 0.05). This is because 80% of softwoods were planted in boreal region while only 4% of hardwoods were plant in the same region based on our datasets, which in turn supports the relationships of C: N ratio with temperature and latitude (Fig. 2d–f).

### N-C scaling patterns after afforestation

Despite the relative complexity and the significant spatial differences of soil medium, N in general scaled isometrically with respect to C over the age sequence, among different climate zones, previous land use types, and plantation species following afforestation (Fig. 3), and in both the organic and the mineral soil horizons (Table 1; Fig. 4). This indicates that isometric patterns did occur temporally and spatially following afforestation through N accumulation in proportion to accrual of C^4^,^14^,^50^. In contrast, the scaling slope of the relationship between N and C in boreal region was larger than 1.0 following afforestation (Fig. 3b). This observation may reflect plants’ exhibition of a different N-C scaling pattern from the general one observed in this (Fig. 3) and other studies^4^,^11^. Theoretically, the size of the scaling slope between N and C should be consistent with the changes in C: N ratio^51^. A higher scaling slope (>1.0) should result from the decreasing C: N ratio probably via the loss of C due to the vulnerability of the organic carbon to climate warming in boreal regions^52^. Interestingly, though C: N ratio revealed significant differences between the organic and the mineral horizons (Figs 1 and 2) and among some of the different groups within the organic and the mineral horizons (Fig. 2), the N-C scaling slopes did not differ significantly (Table 1; Fig. 3). This may be ascribed to different intercepts of the isometric relationships between N and C among them, which was also previously found^11^. The limitation of our synthesis is using data from published studies may not be exactly representative of the actual global afforestation patterns. However, given that the C: N ratios and scaling relationships we found were consistent in spite of any limitations or bias in the data, it is support for the robustness of the C-N patterns following afforestation.

In summary, this synthesis, to our best knowledge, is the first comprehensive analyses of C: N stoichiometry following afforestation. For the organic soil horizon, C: N ratios decreased over the age sequence after afforestation, which may be ascribed to the increasing decomposition of organic matter. C: N ratios increased with increasing MAT (decreasing latitude). Land use types prior to afforestation and plant species also influenced C: N ratios because of the differences in litter quality. For the mineral horizon, the C: N ratio is relatively constant over the age sequence and among land use types and plantation species following afforestation, probably due to the deep assimilation of organic matter by microbes. In contrast to the organic horizon, C: N ratios in the mineral horizon decreased with increasing MAT (decreasing latitude). The N-C isometric pattern in general held true in both the organic and the mineral horizons over the age sequence and among different climate zones, land use types, and plantation species after afforestation. Our analyses indicate the existence of global patterns in C: N ratios in relation to time, temperature, and latitudinal gradients following afforestation. Under global change scenarios, such as warming and N deposition, future studies that investigate how C: N stoichiometry responds to global environmental changes are necessary to advancing our understanding of the effectiveness of C sequestration via afforestation and supporting policy-making.

## Additional Information

**How to cite this article**: Xu, X. *et al.* Carbon: nitrogen stoichiometry following afforestation: a global synthesis. *Sci. Rep.* **6**, 19117; doi: 10.1038/srep19117 (2016).

## Supplementary Material

Supplementary Information

## Figures and Tables

**Table 1:** Summary of reduced major axis (RMA) analysis of the log-transformed N-C stoichiometric relationships for the organic and the mineral soil horizons.

Soil horizon	Slope	95% CI of slope	*r*^2^	*P*	n
Organic horizon	1.08	0.97, 1.20	0.90	<0.001	41
Mineral horizon	0.99	0.92, 1.06	0.75	<0.001	203
