# Supplementary material for: Carbon: nitrogen stoichiometry following afforestation: a global synthesis
Source: Sci Rep. 2016 Jan 8;6:19117. doi: 10.1038/srep19117 (PMC4705480; doi:10.1038/srep19117)
Supplement: Supplementary Information [file srep19117-s1.doc]

**Carbon : nitrogen stoichiometry following afforestation: a global synthesis**

**Running Title:** C : N stoichiometry following afforestation

Xia Xu, Dejun Li, Xiaoli Cheng, Honghua Ruan, Yiqi Luo

**Table S1 Individual studies included in this study. Part I: Organic layer**

| References | Location | Climate | Previous land use | Species | Age, year |
| --- | --- | --- | --- | --- | --- |
| Alberti et al., 2008 | Italy | TM | Gr | Hardwoods | 40-75 |
| Chen et al., 2000 | New Zealand | TM | Gr | Pine | 19 |
| Garten et al., 2002 | USA | STR | Cr | Pine, Hardwoods | 5-11 |
| Grünzweig et al., 2007 | Israel | STR | Pa | Pine | 35 |
| Hooker and Compton, 2003 | USA | TM | Cr | Hardwoods | 47-115 |
| Jug et al., 1999 | Germany | TC | Cr | Hardwoods | 9 |
| Kirschbaum et al., 2008 | New Zealand | TM | Pa | Pine | 18 |
| Mao et al., 2010 | China | TC | Cr | Hardwoods | 5-20 |
| Maquere et al., 2008 | Brazil | TR | Pa | Eucalyptus | 60 |
| Morris et al., 2007 | USA | TC | Cr | Pine, Hardwoods | 53-50 |
| Parfitt et al., 1997 | New Zealand | TM | Pa | Pine | 20 |
| Parﬁtt et al., 2003 | New Zealand | TM | Gr | Pine | 22 |
| Poulton et al., 2003 | UK | TM | Cr | Hardwoods | 82-116 |
| Rosenqvist et al., 2010 | Sweden | BO | Cr | Softwoods | 15-88 |
| Ross et al., 1999 | New Zealand | TM | Pa | Pine | 19 |
| Sharrow and Ismail, 2004 | USA | STR | Pa | Softwoods | 11 |

Note: TM, temperate maritime; STR, subtropical; TC, temperate continental; TR, tropical; BO, boreal; Pa, pasture; Cr, cropland; Gr, natural grassland. Tree species planted were categorized into pine, Eucalyptus, hardwoods (excluding Eucalyptus), and softwoods (excluding pine).

**Table S1** (continued) Individual studies included in this study. Part II: mineral layer

| References | location | climate | Previous  land use | Planted Species | Years after  Afforestation | Sampling depth  (cm) |
| --- | --- | --- | --- | --- | --- | --- |
| Alfredsson et al., 1998 | New Zealand | TM | Pa | Softwoods | 17 | 0-30 |
|  | New Zealand | TM | Pa | Pine | 15 | 0-30 |
| Arevalo et al., 2009 | Canada | BO | Cr | Hardwoods | 9 | 0-50 |
| Chen et al., 2000 | New Zealand | TM | Pa | Pine | 19 | 0-30 |
| Davis, 1994 | New Zealand | TM | Pa | Pine | 10 | 0-10 |
| Del Galdo et al., 2003 | Italy | TM | Cr | Softwoods | 20 | 0-30 |
|  | Italy | TM | Gr | Softwoods | 20 | 0-30 |
| de Koning et al., 2003 | Ecuador | TR | Pa | Hardwoods | 7-30 | 0-50 |
| Farley et al., 2004 | Ecuador | TM | Gr | Pine | 7.5-22.5 | 0-10 |
| Garten et al., 2002 | USA | STR | Cr | Pine | 5-10 | 0-40 |
|  | USA | STR | Cr | Hardwoods | 11 | 0-40 |
| Giddens et al., 1997 | New Zealand | TM | Pa | Pine | 13-30 | 0-10 |
| Groenendijk et al., 2002 | New Zealand | TM | Pa | pine | 17-19 | 0-100 |
| Grünzweig et al., 2007 | Israel | STR | Pa | Pine | 35 | 0-50 |
| Guo et al., 2007 | Australia | TM | Pa | Pine | 16 | 0-100 |
| Huygens et al., 2005 | Chile | TM | Pa | pine | 16 | 0-30 |
| Jackson et al., 2002 | USA | TC | Gr | Hardwoods | 30-90 | 0-100 |
| Jobbagy and Jackson, 2003 | Argentina | TC | Gr | Eucalyptus | 11-95 | 0-20 |
| Jug et al., 1999 | Germany | TC | Cr | Hardwoods | 7,9 | 0-30 |
| Lemenih et al., 2004 | Ethiopia | TR | Cr | Eucalyptus | 17 | 0-20 |
| Liao et al., 2006 | USA | STR | Pa | Hardwoods | 14-132 | 0-15 |
| Ma and Zeng, 2010 | China | TC | Cr | Hardwoods | 5-20 | 0-100 |
|  | China | TC | Cr | Hardwoods | 5-20 | 0-15 |
| Maquere et al., 2008 | Brazil | TR | Pa | Eucalyptus | 60 | 0-100 |
| Menyailo et al., 2002 | Russia | BO | Gr | Softwoods | 28 | 0-30 |
|  | Russia | BO | Gr | Hardwoods | 28 | 0-30 |
|  | Russia | BO | Gr | Pine | 28 | 0-30 |
| Morris et al., 2007 | USA | TC | Cr | Pine | 50 | 0-100 |
|  | USA | TC | Cr | Hardwoods | 53 | 0-100 |
| Parfitt et al., 1997 | New Zealand | TM | Pa | Pine | 20 | 0-20 |
| Parﬁtt et al., 2003 | New Zealand | TM | Pa | Pine | 22 | 0-10 |
| Paul et al., 2003 | Canada | TC | Cr | Hardwoods | 41 | 0-41 |
|  | Canada | TC | Cr | pine | 41 | 0-41 |
|  | USA | TC | Cr | Hardwoods | 100 | 0-100 |
|  | USA | TC | Cr | pine | 100 | 0-100 |
| Pinno and Bélanger, 2008 | Canada | BO | Pa | Softwoods | 50 | 0-40 |
| Poulton et al., 2003 | UK | TM | Cr | Hardwoods | 21-118 | 0-69 |
| Ramirez et al., 2011 | USA | TC | Cr | Pine | 35 | 0-30 |
| Ritter, 2007 | Iceland | BO | Pa | Softwoods | 14-97 | 0-20 |
| Rosenqvist et al., 2010 | Sweden | BO | Cr | Softwoods | 7-88 | 0-25 |
| Ross et al., 1999 | New Zealand | TM | Pa | Pine | 19 | 0-20 |
| Scharenbroch et al., 2010 | USA | TC | Gr | Pine | 44 | 0-38 |
| Sharrow and Ismail, 2004 | USA | STR | Pa | Softwoods | 11 | 0-45 |
| Ussiri et al., 2006 | USA | TC | Pa | Pine | 10 | 0-50 |
|  | USA | TC | Pa | Hardwoods | 10 | 0-50 |
| Wang et al., 2011 | China | STR | Gr | Pine | 19 | 0-100 |
|  | China | STR | Gr | Softwoods | 19 | 0-116 |
| Wei et al., 2009 | China | TC | Gr | Pine | 28 | 0-100 |
| Yan et al., 2009 | China | TC | Pa | Hardwoods | 32 | 0-30 |
| Yeates et al., 2000 | New Zealand | TM | Pa | Pine | 6-25 | 0-10 |
| Zhao et al., 2007 | China | TC | Gr | Pine | 15-30 | 0-20 |
| Zhao et al., 2008 | China | TC | Pa | Pine | 22 | 0-20 |
| Zou and Bashkin, 1998 | USA | TR | Cr | Eucalyptus | 10 | 0-25 |

Note: TM, temperate maritime; BO, boreal; TR, tropical; STR, subtropical; TC, temperate continental; Gr, natural grassland; Pa, pasture; Cr, cropland. Tree species planted were categorized into pine, Eucalyptus, hardwoods (excluding Eucalyptus), and softwoods (excluding pine).

**Notes S1** A list of papers from which data are extracted for this study.

**Alberti G, Peressotti A, Piussi P, Zerbi G. 2008.** Forest ecosystem carbon accumulation during a secondary succession in the Eastern Prealps of Italy. *Forestry* **81**: 1-11.

**Alfredsson H, Condron LM, Clarholm M, Davis MR. 1998.** Changes in soil acidity and organic matter following the establishment of conifers on former grassland in New Zealand. *Forest Ecology and Management* **112** 245-252.

**Arevalo CBM, Bhatti JS, Chang SX, Sidders D. 2009.** Ecosystem carbon stocks and distribution under different land-uses in north central Alberta, Canada. *Forest Ecology and Management* **257** 1776-1785.

**Chen C, Condron L, Davis M, Sherlock R. 2000.** Effects of afforestation on phosphorus dynamics and biological properties in a New Zealand grassland soil. *Plant and Soil* **220**: 151-163.

**Davis MR. 1994.** Topsoil properties under tussock grassland and adjoining pine forest in Otago, New Zealand. *New Zealand Journal of Agricultural Research* **37**: 465-469.

**de Koning GHJ, Veldkamp E, López-Ulloa M. 2003.** Quantification of carbon sequestration in soils following pasture to forest conversion in northwestern Ecuador. *Global Biogeochem. Cycles* **17**(4): 1098.

**Del Galdo I, Six J, Peressotti A, Cotrufo MF. 2003.** Assessing the impact of land-use change on soil C sequestration in agricultural soils by means of organic matter fractionation and stable C isotope. *Global Change Biology* **9**: 1204-1213..

**Farley K, Kelly E, Hofstede R. 2004.** Soil organic carbon and water retention following conversion of grasslands to pine plantations in the Ecuadoran Andes. *Ecosystems* **7**: 729-739.

**Garten C. 2002.** Soil carbon storage beneath recently established tree plantations in Tennessee and South Carolina, USA. *Biomass Bioenerg* **23**: 93-102.

**Giddens K, Parﬁtt R, Percival H. 1997.** Comparison of some soil properties under Pinus radiata and improved pasture. *New Zealand Journal of Agricultural Research* **40**: 409-416.

**Groenendijk FM, Condron LM, Rijkse WC. 2002.** Effects of afforestation on organic carbon, nitrogen and sulfur concentrations in New Zealand hill country soils. *Geoderma* **108** 91- 100.

**Grünzweig J, Gelfand I, Fried Y, Yakir D. 2007.** Biogeochemical factors contributing to enhanced carbon storage follow- ing afforestation of a semi-arid shrubland. *Biogeosciences* **4**: 891-904.

**Guo L, Wang M, Gifford R. 2007.** The change of soil carbon stocks and ﬁne root dynamics after land use change from a native pasture to a pine plantation. *Plant and Soil* **299**: 251-262.

**Hernandez-Ramirez G, Sauer TJ, Cambardella CA, Brandle JR, James DE. 2011.** Carbon Sources and Dynamics in Afforested and Cultivated Corn Belt Soils. *Soil Sci. Soc. Am. J.* **75**(1): 216-225.

**Hooker TD, Compton JE. 2003.** Forest ecosystem carbon and nitrogen accumulation during the first century after agricultural abandonment. *Ecological Applications* **13**(2): 299-313.

**Hughes RF, Kauffman JB, Jaramillo VJ. 1999.** Biomass, carbon, and nutrient dynamics of secondary forests in a humid tropical region of Mexico. *Ecology* **80**(6): 1892-1907.

**Huygens D, Boeckx P, Cleemput OV, Oyarzun C, Godoy R. 2005.** Aggregate and soil organic carbon dynamics in South Chilean Andisols. *Biogeosciences* **2**: 159-174.

**Jackson RB, Banner JL, Jobbagy EG, Pockman WT, Wall DH. 2002.** Ecosystem carbon loss with woody plant invasion of grasslands. *Nature* **418**: 623-626.

**Jobbagy EG, Jackson RB. 2003.** Patterns and mechanisms of soil acidification in the conversion of grasslands to forests. *Biogeochemistry* **54**: 205-229.

**Jug A, Makeschin F, Rehfuess K, Hofmann-Schielle C. 1999.** Short-rotation plantations of balsam poplars, aspen and willows on former arable land in the Federal Republic of Germany. III. Soil ecological effects. *Forest Ecology and Management* **121**: 85-99.

**Kirschbaum MUF, Guo LB, Gifford RM. 2008.** Why does rainfall affect the trend in soil carbon after converting pastures to forests?: A possible explanation based on nitrogen dynamics. *Forest Ecology and Management* **255**(7): 2990-3000.

**Lemenih M, Olsson M, Karltun E. 2004.** Comparison of soil attributes under Cupressus lusitanica and Eucalyptus saligna established on abandoned farmlands with continuously cropped farmlands and natural forest in Ethiopia. *Forest Ecology and Management* **195** 57-67.

**Liao JD, Boutton TW, Jastrow JD. 2006.** Storage and dynamics of carbon and nitrogen in soil physical fractions following woody plant invasion of grassland. *Soil Biology & Biochemistry* **38**: 3184-3196.

**Mao R, Zeng D. 2010.** Changes in Soil Particulate Organic Matter, Microbial Biomass, and Activity Following Afforestation of Marginal Agricultural Lands in a Semi-Arid Area of Northeast China. *Environmental Management* **46**: 110-116.

**Mao R, Zeng D-H, Hu Y-L, Li L-J, Yang D. 2010.** Soil organic carbon and nitrogen stocks in an age-sequence of poplar stands planted on marginal agricultural land in Northeast China. *Plant and Soil* **332**: 277-287.

**Maquere V, J.P.LACLAU, M.BERNOUX, L.SAINT-ANDRE, J.L.M.GONCALVE, CERRI CC, M.C.PICCOLO, J.RANGER. 2008.** Inﬂuence of land use (savanna, pasture, Eucalyptus plantations) on soil carbon and nitrogen stocks in Brazil. *European Journal of Soil Science* **59**: 863-877.

**Menyailo OV, Hungate BA, Zech W. 2002.** Tree species mediated soil chemical changes in a Siberian artiﬁcial afforestation experiment-tree species and soil chemistry. *Plant and Soil* **242**: 171-182.

**Morris S, Bohm S, Haile-Mariam S, Paul E. 2007.** Evaluation of carbon accrual in afforested agricultural soils. *Global Change Biology* **13**: 1145-1156.

**Ouimet R, Tremblay S, Perie C, Pregent G. 2007.** Ecosystem carbon accumulation following fallow farmland afforestation with red pine in southern Quebec. *Canadian Journal of Forest Research-Revue Canadienne De Recherche Forestiere* **37**(6): 1118-1133.

**Parﬁtt R, Percival H, Dahlgren R, Hill L. 1997.** Soil and solution chemistry under pasture and radiata pine in New Zealand. *Plant and Soil* **191**: 279-290.

**Parﬁtt RL, Scott NA, Ross DJ, Salt GJ, Tate KR. 2003.** Land-use change effects on soil C and N transformations in soils of high N status: comparisons under indigenous forest, pasture and pine plantation. *Biogeochemistry* **66**: 203-221.

**Paul EA, Morris SJ, Six J, Paustian K, Gregorich EG. 2003.** Interpretation of Soil Carbon and Nitrogen Dynamics in Agricultural and Afforested Soils. *Soil Sci. Soc. Am. J.* **67**(5): 1620-1628.

**Pérez-Cruzado C, Mansilla-Salinero P, Rodríguez-Soalleiro R, Merino A.** Influence of tree species on carbon sequestration in afforested pastures in a humid temperate region. *Plant and Soil*: 1-21.

**Pérez-Cruzado C, Mansilla-Salinero P, Rodríguez-Soalleiro R, Merino A. 2011.** Influence of tree species on carbon sequestration in afforested pastures in a humid temperate region. *Plant and Soil*: 1-21.

**Pinno B, Belanger N. 2008.** Ecosystem carbon gains from afforestation in the Boreal Transition ecozone of Saskatchewan (Canada) are coupled with the devolution of Black Chernozems. *Agriculture Ecosystems and Environment* **123**: 56-62.

**Poulton PR, Pye E, Hargreaves PR, Jenkinson DS. 2003.** Accumulation of carbon and nitrogen by old arable land reverting to woodland. *Global Change Biology* **9**: 942-955.

**Ramirez GH, Sauer TJ, Cambardella CA, Brandle JR, James DE. 2011** Carbon Sources and Dynamics in Afforested and Cultivated US Corn Belt Soils. *Soil Science Society of America Journal* **75**: doi:10.2136/sssaj2010.0114.

**Ritter E. 2007.** Carbon, nitrogen and phosphorus in volcanic soils following afforestation with native birch (*Betula pubescens*) and introduced larch (*Larix sibirica*) in Iceland. *Plant and Soil* **295**: 239-251.

**Rosenqvist L, Kleja DB, Johansson M-B. 2010.** Concentrations and ﬂuxes of dissolved organic carbon and nitrogen in a Picea abies chronosequence on former arable land in Sweden. *Forest Ecology and Management* **259** 275-285.

**Ross D, Tate K, Scott N, Feltham C. 1999.** Land-use change: effects on soil carbon, nitrogen and phosphorus pools and ﬂuxes in three adjacent ecosystems. *Soil Biology and Biochemistry* **31**: 803-813.

**Scharenbroch B, Flores-Mangual M, Lepore B, Bockheim J, Lowery B. 2010.** Tree Encroachment Impacts Carbon Dynamics in a Sand Prairie in Wisconsin. *Soil Sci Soc Am J* **74**: 956-968.

**Sharrow SH, Ismail S. 2004.** Carbon and nitrogen storage in agroforests, tree plantations, and pastures in western Oregon, USA. *Agroforestry Systems* **60**: 123-130.

**Thuille A, Buchmann N, Schulze ED. 2000.** Carbon stocks and soil respiration rates during deforestation, grassland use and subsequent Norway spruce afforestation in the Southern Alps, Italy. *Tree Physiology* **20**(13): 849-857.

**Tremblay S, Périé C, Ouimet R. 2006.** Changes in organic carbon storage in a 50 year white spruce plantation chronosequence established on fallow land in Quebec. *Canadian Journal of Forest Research* **36**(11): 2713-2723.

**Ussiri DAN, Lal R, Jacinthe PA. 2006.** Soil properties and carbon sequestration of afforested pastures in reclaimed minesoils of Ohio. *Soil Science Society America Journal* **70**: 1797-1806.

**Vesterdal L, Ritter E, Gundersen P. 2002.** Change in soil organic carbon following afforestation of former arable land. *Forest Ecology and Management* **169**(1-2): 137-147.

**WANG S, LIU J, ZHANG C, YI C, WU W. 2011.** Effects of afforestation on soil carbon turnover in China’s subtropical region. *J. Geogr. Sci.* **21**: 118-134.

**Wei X, Shao M, Fu X, Horton R, Li Y, Zhang X. 2009.** Distribution of soil organic C, N and P in three adjacent land use patterns in the northern Loess Plateau, China. *Biogeochemistry* **96**: 149-162.

**Yan J, Zhu X, Zhao J. 2009.** Effects of grassland conversion to cropland and forest on soil organic carbon and dissolved organic carbon in the farming-pastoral ecotone of Inner Mongolia. *Acta Ecologica Sinica* **29**: 150-154.

**Yeates GW, Hawke MF, Rijkse WC. 2000.** Changes in soil fauna and soil conditions under Pinus radiata agroforestry regimes during a 25-year tree rotation. *Biology and Fertility of Soils* **31**: 391-406.

**Zhao Q, Zeng DH, Fan ZP, Lee DK. 2008.** Effect of land cover change on soil phosphorus fractions in southeastern Horqin sandy land, northern China. *Pedosphere* **18**: 741-748.

**Zhao Q, Zeng DH, Lee DK, He XY, Fan ZP, Jin YH. 2007.** Effects of Pinus sylvestris var. mongolica afforestation on soil phosphorus status of the Keerqin Sandy Lands in China. *Journal of Arid Environments* **69** 569-582.

**Zou X, Bashkin M. 1998.** Soil carbon accretion and earthworm recovery following revegetation in abandoned sugarcane ﬁelds. *Soil Biology and Biochemistry* **30**: 825-830.
